# Injecting drug use and hepatitis C virus infection independently increase biomarkers of inflammatory disease risk which are incompletely restored by curative direct-acting antiviral therapy

**DOI:** 10.3389/fimmu.2024.1352440

**Published:** 2024-02-14

**Authors:** Anna C. Hearps, Nikil Vootukuru, Salimeh Ebrahimnezhaddarzi, Brendan L. Harney, Irene Boo, Long Nguyen, Damian Pavlyshyn, Paul M. Dietze, Heidi E. Drummer, Alexander J. Thompson, Anthony Jaworowski, Margaret E. Hellard, Rachel Sacks-Davis, Joseph S. Doyle

**Affiliations:** ^1^ Disease Elimination Program, Burnet Institute, Melbourne, VIC, Australia; ^2^ Department of Infectious Diseases, The Alfred and Monash University, Melbourne, VIC, Australia; ^3^ Department of Gastroenterology, Eastern Health and Monash University, Melbourne, VIC, Australia; ^4^ School of Public Health and Preventive Medicine, Monash University, Melbourne, VIC, Australia; ^5^ National Drug Research Institute, Curtin University, Melbourne, VIC, Australia; ^6^ The Peter Doherty Institute for Infection and Immunity, University of Melbourne, Melbourne, VIC, Australia; ^7^ Department of Microbiology, Monash University, Melbourne, VIC, Australia; ^8^ Department of Gastroenterology, St Vincent’s Hospital and the University of Melbourne, Melbourne, VIC, Australia; ^9^ Melbourne School of Population and Global Health, The University of Melbourne, Melbourne, VIC, Australia

**Keywords:** people who inject drugs, hepatitis C virus, inflammation, direct-acting antivirals, biomarkers

## Abstract

**Background:**

Hepatitis C virus (HCV) infections are more prevalent in people who inject drugs (PWID) who often experience additional health risks. HCV induces inflammation and immune alterations that contribute to hepatic and non-hepatic morbidities. It remains unclear whether curative direct acting antiviral (DAA) therapy completely reverses immune alterations in PWID.

**Methods:**

Plasma biomarkers of immune activation associated with chronic disease risk were measured in HCV-seronegative (n=24) and HCV RNA+ (n=32) PWID at baseline and longitudinally after DAA therapy. Adjusted generalised estimating equations were used to assess longitudinal changes in biomarker levels. Comparisons between community controls (n=29) and HCV-seronegative PWID were made using adjusted multiple regression modelling.

**Results:**

HCV-seronegative PWID exhibited significantly increased levels of inflammatory biomarkers including soluble (s) TNF-RII, IL-6, sCD14 and sCD163 and the diabetes index HbA1c as compared to community controls. CXCL10, sTNF-RII, vascular cell adhesion molecule-1 and lipopolysaccharide binding protein (LBP) were additionally elevated in PWID with viremic HCV infection as compared to HCV- PWID. Whilst curative DAA therapy reversed some biomarkers, others including LBP and sTNF-RII remained elevated 48 weeks after HCV cure.

**Conclusion:**

Elevated levels of inflammatory and chronic disease biomarkers in PWID suggest an increased risk of chronic morbidities such as diabetes and cardiovascular disease. HCV infection in PWID poses an additional disease burden, amplified by the incomplete reversal of immune dysfunction following DAA therapy. These findings highlight the need for heightened clinical surveillance of PWID for chronic inflammatory diseases, particularly those with a history of HCV infection.

## Background

Chronic hepatitis C virus (HCV) infection is a major public health issue (1), with approximately 58.6 million people living with chronic HCV and an estimated 1.5 million new infections each year (1, 2). HCV primarily infects liver hepatocytes causing inflammation and liver damage leading to morbidities including liver cirrhosis and hepatocellular carcinoma (3). HCV also causes extrahepatic manifestations including increased risk of non-hodgkins lymphoma, kidney and rheumatic disease, type 2 diabetes and cardiovascular disease (4, 5). These extrahepatic morbidities are likely mediated at least in part by systemic activation of circulating immune cells and inflammation in response to viral particles and antigens during viremia, as well as cellular damage resulting from virus infection. Similar to HCV, chronic HIV infection is also associated with increased risk of multiple inflammatory diseases (6) and this risk persists despite viral suppression with antiretroviral therapy, implying some impacts of chronic viral disease are not fully reversed upon suppression of viral replication.

HCV can now be cured with direct acting antiviral (DAA) therapy, which provides substantial reductions in both hepatic and non-hepatic sequelae of HCV (5). However, people treated for HCV with interferon-free DAA therapy continue to experience substantially higher rates of all-cause mortality after HCV cure, ranging from 3-14 times higher than the general population depending on the extent of liver disease at the time of treatment (7). It is thus unclear whether DAA therapy completely reverses HCV-related pathologies that may contribute to increased risk of both hepatic and extrahepatic morbidities. Understanding systemic HCV-associated changes which persist post-cure are highly relevant for the ongoing clinical management of individuals with a history of HCV infection.

Inflammation is a key pathogenic mechanism of liver damage during HCV infection and may also drive extrahepatic comorbidities such as cardiovascular disease which are more prevalent in people with HCV (reviewed in (5)). Chronic HCV infection is associated with systemic immune activation and elevated levels of interferons, inflammatory biomarkers including IL-6 and TNF/TNF receptors, chemokines such as CXCL10 and markers of myeloid cell activation including soluble (s) markers sCD163 and sCD14 (8–13). Conflicting data exist regarding the impact of HCV elimination on reversal of immune activation; whilst some biomarkers including CXCL10, IL-10 and sCD163 are consistently reduced following HCV elimination (8, 9, 13–18), others including IL-6, TNF and sCD14 are unaffected or inconsistently altered by DAA therapy (9, 13–16). Importantly, numerous studies suggest that DAA therapy does not restore levels of many parameters including sCD163 and CXCL10 to those found in comparable, HCV-seronegative populations (8, 13, 14, 17–19). Chronic inflammation is associated with increased risk of a number of chronic morbidities and mortality in both the general population and in the setting of HIV and HCV infection (20–23). Therefore, understanding whether elements of HCV-associated inflammation persist post DAA therapy is relevant for the long-term clinical management of individuals after HCV cure.

In most high-income countries, the HCV epidemic is concentrated in populations of people who inject drugs (PWID) who represent up to 80% of new HCV infections in many countries (24). PWID experience additional health risks associated with injecting drug use (25–27) and behavioural factors such as tobacco smoking, alcohol use and diet, which increase chronic inflammation (28, 29), may further contribute to poor health. The additional burden of HCV in PWID may therefore potentiate inflammatory diseases and/or compromise the restorative potential of DAA-mediated HCV cure on immune function. However, to date there is limited information regarding the discrete effects of HCV and substance use-related risks on inflammatory pathways in PWID populations who are disproportionately affected by HCV.

In this study we aimed to delineate the independent effects of both HCV infection and injecting drug use on soluble inflammatory and immune activation biomarkers among people with HCV who inject drugs. Furthermore, we investigated whether these biomarkers normalised to levels comparable to HCV-uninfected PWID after curative DAA therapy.

## Materials and methods

### Study design

#### Cohorts and participants

This retrospective cohort study included a total of 85 participants in three distinct groups (1); participants with no history of injecting drug use or HCV infection (general community controls; “CC”, n=29); (2) participants with a history of injecting drug use but not HCV infection (“PWID/HCV-”, n=24) and (3) participants with a history of injecting drug use and current HCV infection (“PWID/HCV+”, n=32). Participants in the PWID groups were selected from three prospective cohort studies (Networks 2, SuperMIX and TAP), which all included PWID recruited from Melbourne, Australia. These studies involved regular study visits which included structured interviews and blood collection (30–32). PWID participants were primarily Australian-born with approximately 80% reporting recent heroin use, with a minority of participants reporting meth/amphetamine or pharmaceutical opiate use (30–32).

#### Inclusion/exclusion criteria

A history of ever using injecting drugs was an inclusion criterion for both PWID cohorts. PWID/HCV- participants were defined as those with a negative HCV antibody test and were included if they were comparable in age (within 5 years) and sex to those in the PWID/HCV+ group. Inclusion criteria for PWID/HCV+ participants were being HCV antibody and RNA positive at baseline, completion of 12 weeks of DAA therapy with sofosbuvir/velpatasvir, achieving sustained viral response after 12 weeks of DAA therapy, maintaining undetectable HCV RNA at all post-DAA timepoints up to and including 48 weeks and the availability of stored samples at the required timepoints. CC were volunteers from the general community with no self-reported history of acute or chronic disease (including HCV infection) or injecting drug use (33, 34). CC were sex-matched and of a comparable age (within 5 years) to PWID groups. The study was approved by the Alfred Hospital Research Ethics Committee (approval #40/18).

#### Analysis timepoints

Samples from PWID/HCV+ participants were analysed at baseline (prior to initiating DAA therapy; “pre-treatment”), after 12 weeks of DAA when HCV viremia was undetectable (end of treatment; “EOT”) and 48 weeks after DAA initiation (“48wk post-DAA”). Due to sample availability, the EOT timepoint analysis included samples taken at 8 (n=2), 12 (n=29) or 28 (n=1) weeks after DAA commencement whilst the 48wk post-DAA timepoint included samples taken at 48 (n=29), 60 (n=2) or 68 (n=1) weeks post DAA commencement. Pre-HCV infection samples were available for some individuals in the PWID/HCV+ group which were also included in the analysis. HCV genotypes were not determined here, however a previous analysis of PWID populations from which these samples were obtained reported a predominance of genotype 1 (45%) and 3 (42%, primarily 3a) (30), consistent with other reports regarding HCV genotype prevalence in Australia (35, 36). Participants in the CC and PWID/HCV- groups were analysed at a single timepoint.

### Data collection, blood sampling and biomarker analysis

For both PWID groups, socio-demographical, behavioural (including drug injecting frequency) and clinical (HCV antibody, HCV RNA, platelets, white cell count, alanine/aspartate transaminase [ALT/AST], bilirubin, albumin, protein) data were obtained from the Networks 2, TAP and SuperMIX databases. Available data for the CC group was limited to age, sex and self-reported medical history.

Cryopreserved plasma samples for each group were used to measure plasma levels of immune biomarkers relevant to chronic disease and/or known to be altered by HCV including markers of (1) immune activation (CXCL10), (2) endothelial activation (soluble vascular cell adhesion molecule-1 [VCAM]), (3) inflammation (soluble TNF-receptor II [sTNF-RII], IL-6, high sensitivity C-reactive protein [hsCRP]), (4) coagulation (d-dimer) and (5) myeloid cell activation (sCD14, sCD163 and lipopolysaccharide binding protein [LBP]) using customised human magnetic Luminex assays (R&D Systems, Minneapolis, MN), high sensitivity Quantikine ELISA (for IL-6; R&D Systems) or quantitative immunoturbidity assay (for CRP; ARCHITECT, Abbott Laboratories, Abbott Park, IL). Levels of glycated haemoglobin (HbA1c; indicative of diabetes risk) were measured by ELISA (Aviva Systems Biology, San Diego, CA).

### Data analysis

All statistical analyses were performed using the R programming environment, version 4.1. Differences between baseline characteristics of the study groups were assessed using Chi-squared and Fisher exact tests for categorical variables and Wilcoxon rank sum and Wilcoxon rank sum exact tests for continuous variables. All inflammatory markers were log-transformed to account for non-normal distribution. Multivariable linear regression models were used to evaluate differences in the log values of each inflammatory and immune activation marker between CC and PWID/HCV- control groups. Models were adjusted for age and sex given the potential effects of these variable on the parameters measured. History of injecting drug use was assessed as a binary exposure variable. Generalised estimating equation models with an autoregressive structure were used to evaluate differences between the log values of each inflammatory marker among PWID/HCV- and PWID/HCV+ by group and DAA-therapy related timepoint (PWID/HCV- group vs PWID/HCV+ group at pre-treatment, end of treatment and 48 weeks post treatment timepoints). Models were adjusted for age (continuous variable), sex (binary variable) and injection frequency (daily injecting drug use as a binary variable; participants with missing data were excluded from these analyses). Statistical significance was assessed at p<0.05 using two-sided p-values.

## Results

### Patient characteristics

Demographic and clinical information for the cohorts utilised in this study are described in **Tables 1**, **2**. Basic demographic information was available for CC participants whilst more detailed information was available for PWID groups. Groups were well matched for age and sex and there were no reported cases of HIV or HBV co-infection. At first included timepoint, all participants in the PWID/HCV- and PWID/HCV+ groups reported a history of injecting drug use; 75% and 100% of HCV- and HCV+ PWID participants reported injecting drug use in the past month and 43% and 42% reported daily injecting drug use over past month (defined as injecting ≥7 times per week), respectively. PWID/HCV+ were more likely to be unemployed but also more likely to be in stable housing as compared to PWID/HCV- (p=0.032 for both in unadjusted analyses, **Table 2**). Liver function biomarkers (ALT and AST) were elevated in PWID/HCV+ (p ≤ 0.001 for both) whilst plasma albumin was slightly lower (p=0.015, **Table 2**).

**Table 1:** Baseline cohort description of community controls and HCV- people who inject drugs (PWID).

Characteristic	PWID (HCV-)	Community Controls	p-value^2^
n	24	29	
Sex			0.650
Female	8 (33%)	8 (28%)	
Male	16 (67%)	21 (72%)	
Age	37 (32, 41) ^1^	40 (33, 47) ^1^	0.386
Unknown	3	0	
Injecting Drugs Monthly	18 (75%)	0 (0%)	<0.001

^1^Median (IQR); n (%).

^2^Pearson's Chi-squared test; Wilcoxon rank sum test; Fisher's exact test.

**Table 2:** Baseline demographics of people who inject drugs (PWID) cohorts.

Characteristic	PWID/HCV-^1^	PWID/HCV+^1^	p-value^2^
n	24	32	
Sex			0.495
Female	8 (33%)	8 (25%)	
Male	16 (67%)	24 (75%)	
Age	37 (32, 41)	39 (34, 47)	0.239
Employment Status			**0.032**
Employed	7 (29%)	2 (6%)	
Unemployed	17 (71%)	29 (94%)	
Unknown	0	1	
Accommodation Status^3^			**0.032**
Stable	17 (71%)	29 (94%)	
Unstable	7 (29%)	2 (6%)	
Unknown	0	1	
Alcohol Use			0.123
Never	7 (32%)	12 (46%)	
Less than 4 times a week	8 (36%)	12 (46%)	
4 or more times a week	7 (32%)	2 (8%)	
Unknown	2	6	
Age Commenced Injecting	16.5 (15.8, 21.2)	18.0 (15.0, 21.0)	0.858
Unknown	0	1	
Injecting drug use – past month	18 (75%)	25 (100%)	**0.010**
Unknown	0	7	
Injecting Drugs Daily	9 (43%)	10 (42%)	0.936
Unknown	3	8	
ALT (U/L)	19 (15, 28)	50 (38, 78)	**<0.001**
Unknown	0	1	
AST (U/L)	24 (21, 36)	43 (35, 62)	**0.001**
Unknown	2	1	
White cell count (x10^9/L)	7.00 (5.32, 8.38)	7.40 (6.20, 8.50)	0.757
Unknown	18	1	
Platelets (x10^9/L)	253 (233, 344)	243 (184, 308)	0.343
Unknown	18	1	
Total Bilirubin (umol/L)	6.0 (5.0, 8.8)	7.0 (4.0, 12.0)	0.435
Unknown	2	1	
Albumin (g/L)	41.0 (39.0, 43.0)	38.0 (37.0, 40.0)	**0.015**
Unknown	2	1	
Total Protein (g/L)	74.0 (69.0, 77.0)	74.0 (71.5, 77.0)	0.949
Unknown	2	1	

^1^Median (IQR); n (%).

^2^Wilcoxon rank sum exact test; Pearson’s Chi-squared test; Fisher’s exact test; Wilcoxon rank sum test. P values were derived from unadjusted analyses.

^3^Stable accommodation included owner occupied, rental and rent free. All other forms of accommodation including boarding, boarding house, squat, homeless/street were defined as unstable.

ALT, alanine transaminase; AST, aspartate transaminase.

Bold values indicate those which are statistically significant (p<0.05).

### People with a history of injecting drug use exhibit elevated levels of a range of biomarkers of inflammation and chronic disease risk

**Figure 1:**
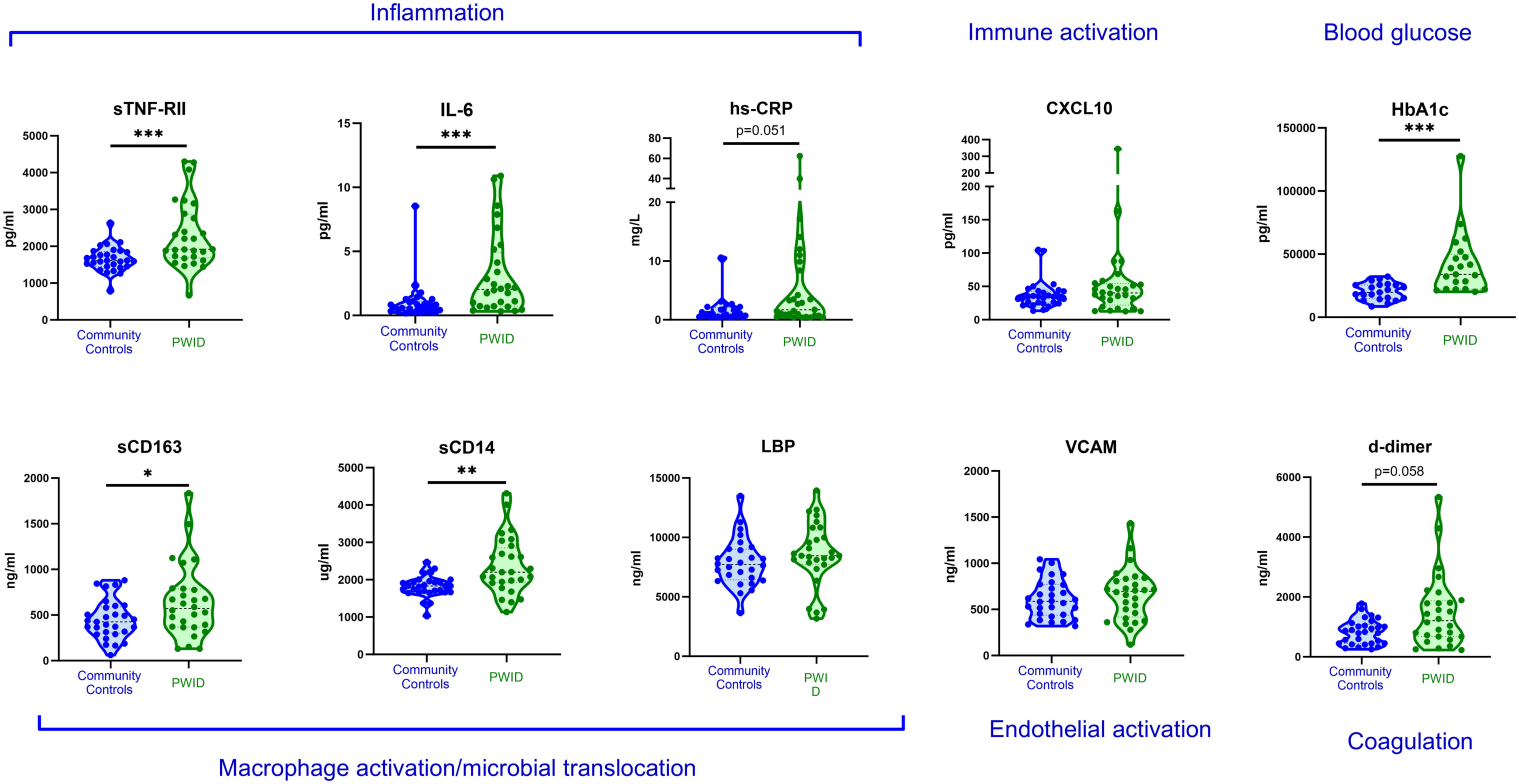
Plasma biomarkers levels in community controls and PWID. Plasma biomarkers of inflammation and chronic disease risk were measured in HCV- community controls and PWID of a comparable age and sex. Annotations in blue text indicate association of each biomarker with relevant biological process. *, ** and *** indicate p value <0.05, 0.01 and 0.001 respectively as determined by multivariable linear regression analysis adjusted for age, sex and recent injecting drug use as described in **Table 3**.

A preliminary analysis was conducted comparing participants in the CC and PWID/HCV- groups to identify any differences in biomarker levels (**Figure 1**). Multivariable linear regression analysis indicated that a lifetime history of injecting drug use was associated with significantly higher levels of multiple inflammatory biomarkers including sTNF-RII and IL-6 (adjusted p<0.001 for both) and the myeloid cell activation markers sCD163 and sCD14 (p=0.048 and 0.003 respectively; **Table 3**, where a negative beta coefficient indicates lower biomarker levels in CC). Plasma levels of glycated haemoglobin (HbA1c), which indicates glycaemic control over the past 3 months and is thus a relevant biomarker for diabetes, were also significantly elevated in PWID/HCV- as compared to the CC group (p<0.001). Elevated levels of the coagulation marker d-dimer and the acute phase inflammatory factor hsCRP in PWID/HCV- did not reach statistical significance (p=0.058 and 0.051 respectively). Age and sex were not significantly related to levels of the abovementioned biomarkers in this analysis.

**Table 3:** Multivariable Linear Regression analysis of inflammatory biomarkers in community controls with reference to PWID.

	Sex: Male		Age (10/years)		History of Injecting Drugs	
	Beta (95% CI)^1^	p-value	Beta (95% CI)^1^	p-value	Beta (95% CI)^1^	p-value
**Log CXCL10**	0.14 (-0.23 to 0.51)	0.437	-0.10 (-0.32 to 0.12)	0.365	-0.12 (-0.47 to 0.22)	0.472
**Log sTNF-RII**	0.02 (-0.15 to 0.19)	0.836	0.04 (-0.06 to 0.14)	0.431	**-0.31 (-0.47 to -0.15)**	**<0.001**
**Log VCAM**	0.10 (-0.17 to 0.37)	0.457	0.01 (-0.14 to 0.17)	0.866	-0.04 (-0.28 to 0.21)	0.774
**Log sCD163**	0.03 (-0.34 to 0.40)	0.860	-0.03 (-0.24 to 0.19)	0.812	**-0.35 (-0.69 to 0.00)**	**0.048**
**Log D-Dimer**	-0.36 (-0.75 to 0.03)	0.073	0.22 (-0.01 to 0.45)	0.064	-0.35 (-0.72 to 0.01)	0.058
**Log sCD14**	-0.09 (-0.25 to 0.07)	0.249	0.03 (-0.07 to 0.12)	0.551	**-0.23 (-0.37 to -0.08)**	**0.003**
**Log LBP**	-0.15 (-0.35 to 0.04)	0.121	0.01 (-0.10 to 0.13)	0.810	-0.02 (-0.20 to 0.16)	0.829
**Log IL-6**	-0.03 (-0.62 to 0.56)	0.924	0.02 (-0.33 to 0.37)	0.901	**-0.98 (-1.5 to -0.43)**	**<0.001**
**Log hsCRP**	-0.26 (-1.0 to 0.50)	0.491	0.19 (-0.25 to 0.64)	0.386	-0.71 (-1.4 to 0.00)	0.051
**Log HbA1c**	-0.07 (-0.40 to 0.27)	0.685	-0.01 (-0.20 to 0.17)	0.905	**-0.64 (-0.93 to -0.34)**	**<0.001**

^1^CI, Confidence Interval.

s, soluble, TNF-RII, tumor necrosis factor receptor II; VCAM, vascular cellular adhesion molecule; LBP, lipopolysaccharide binding protein; IL-6, interlukin-6; hsCRP, high sensitivity C-reactive protein; HbA1c, glycated haemoglobin.

Bold values indicate those which are statistically significant (p<0.05).

To investigate whether recent injecting drug use contributed to the abovementioned differences, regression analyses adjusting for injecting drug using within the past month was performed (also adjusting for age and sex). These analyses indicated individuals who had injected drugs within the previous month exhibited significantly higher levels of sTNF-RII, d-dimer, HbA1c (p<0.01 for all) and sCD14 (p=0.031; **Supplementary Table 1**). These data indicate that this cohort of PWID seronegative for HCV exhibit elevated levels of a range of soluble biomarkers including those associated with increased risk of chronic diseases, and that recent injecting drug use may contribute to elevated levels of some of these factors.

### Viremic HCV infection is associated with elevated levels of biomarkers indicative of immune and endothelial activation

**Figure 2:**
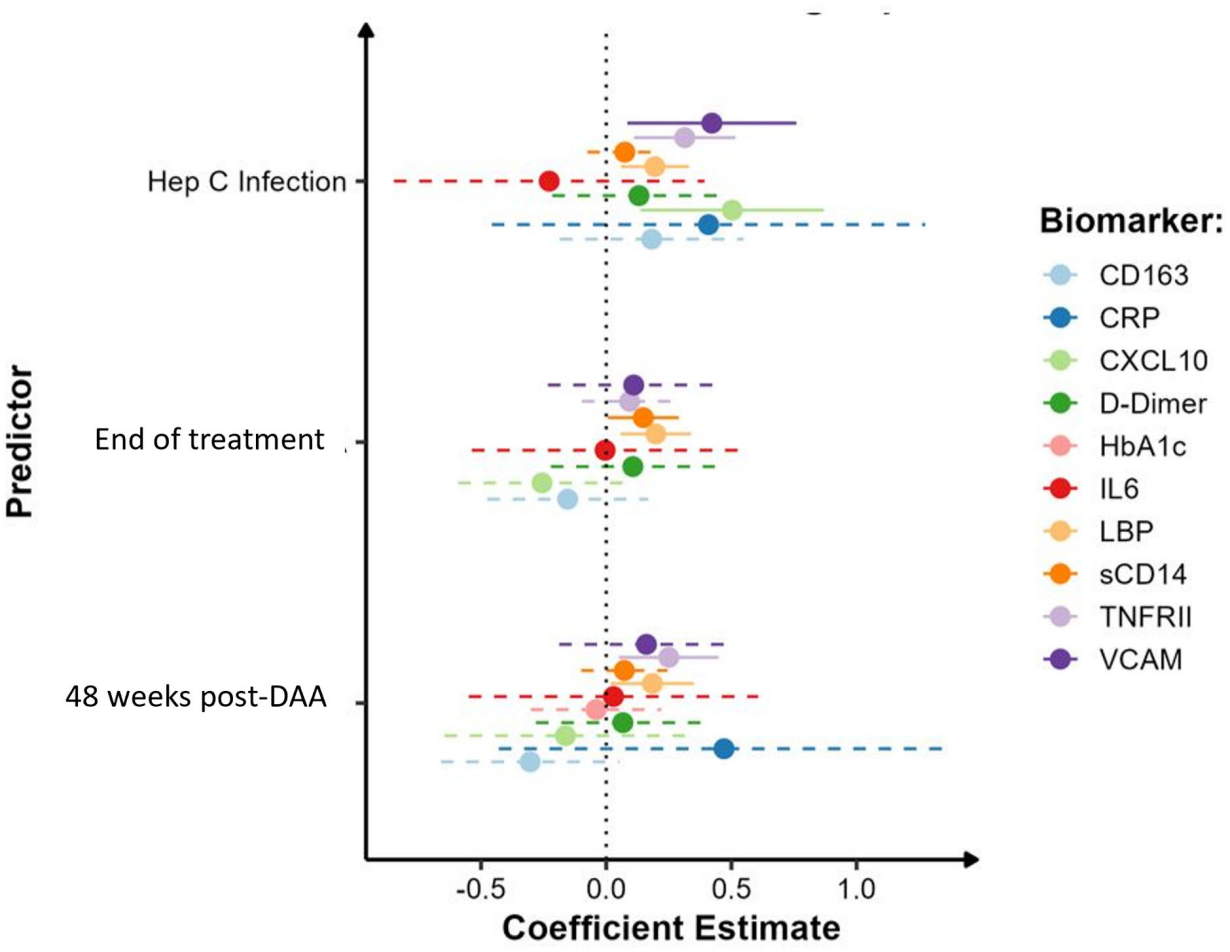
Impact of HCV infection and DAA therapy on biomarker levels in PWID. Model estimates of inflammatory biomarkers by HCV infection (pre-treatment timepoint), at end of DAA treatment and 48 weeks post-DAA therapy in HCV+ PWID with reference to HCV-uninfected PWID. Analysis was adjusted for sex, age and daily injecting drug use. Solid lines indicate parameters which were significantly different to HCV-uninfected PWID.

**Figure 3:**
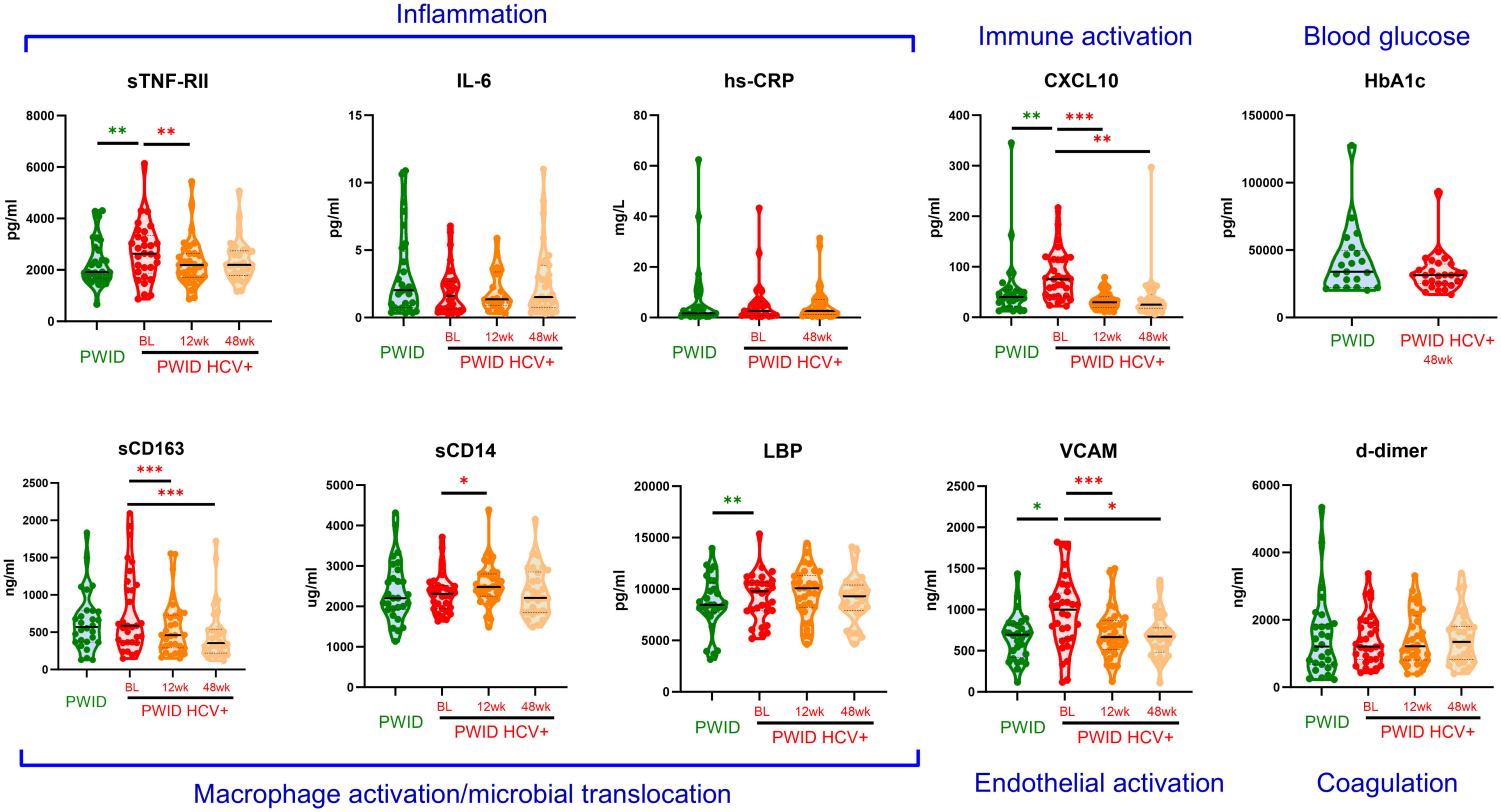
Plasma biomarker levels in PWID and HCV+ PWID pre and post DAA therapy. Inflammatory biomarkers were measured in PWID and PWID with chronic HCV infection prior to treatment (baseline; BL), and 12 weeks (12wk) or 48 weeks (48wk) after curative DAA therapy. Untransformed data are shown. CRP was not assessed at the 12 week timepoint whilst HbA1c was only assessed in HCV+ PWID 48 weeks after DAA therapy. Annotations in blue text indicate the association of each biomarker with relevant biological process. *, ** and *** indicate p value <0.05, 0.01 and 0.001, respectively as determined by multivariable linear regression (green asterisks, comparing to PWID to HCV+ PWID) or generalised estimating equation (red asterisks, comparing post-DAA to baseline in HCV+ PWID) analysis as described in **Table 4** and **Supplementary Table 2**, respectively. Note: similar statistical significance was obtained after removing two outlier datapoints from the CXCL10 analysis (data not shown).

Given the abovementioned differences in biomarker levels in PWID/HCV- as compared to those who did not report having injected drugs, we assessed the specific effect of viremic HCV infection on immune activation biomarkers exclusively in PWID populations. For these analyses, we compared 24 PWID/HCV- (median age [IQR] 37 [32, 41], 33% female, **Table 2**) and 32 PWID with viremic HCV infection at baseline (PWID/HCV+; median age 39 [34, 47]; 25% female) with a median [IQR] viral load of 202,229 [62,237 - 1,987,626] copies/mL at enrolment. Generalised estimating equation models were used adjusting for age, sex and daily injecting drug use (**Table 4**, **Figure 2**). Viremic HCV infection was associated with elevated levels of the immune activation marker CXCL10 (p=0.007), the inflammatory marker sTNF-RII (p=0.002), the endothelial activation marker VCAM (p=0.014) and also the acute phase protein LBP (p=0.005), which binds to bacterial lipopolysaccharide (LPS; **Table 4**, **Figures 2**, **3**). Levels of other biomarkers including IL-6, sCD163, sCD14 and d-dimer which were elevated in PWID/HCV- as compared with CC, were not significantly different in viremic PWID/HCV+ as compared with PWID/HCV- (**Table 4**). Increasing age was associated with higher levels of the blood coagulation biomarker d-dimer but lower levels of HbA1c (p ≤ 0.01 for both, **Table 4**). Sex did not significantly alter any parameter, whilst the association between daily injection frequency and higher d-dimer levels approached statistical significance (p=0.057, data not shown), consistent with observations made comparing PWID/HCV- to CC (**Table 3**). Taken together, these analyses indicate that in addition to increased inflammation identified above to be associated with a history of injecting drug use, PWID with viremic HCV infection exhibit significantly elevated levels of additional biomarkers of inflammation as compared to PWID without HCV.

**Table 4:** Generalised estimating equation analysis of inflammatory biomarkers in PWID/HCV+ pre and post DAA therapy compared to HCV-uninfected PWID.

	Timepoint (Ref: PWID/HCV-)						Age (10/years)		
	Pre-treatment		EOT		48wk post-DAA				
	Beta (95% CI)^1^	p-value	Beta (95% CI)^1^	p-value	Beta (95% CI)^1^	p-value		Beta (95% CI)^1^	p-value
**Log CXCL10**	**0.50 (0.14 to 0.87)**	**0.007**	-0.26 (-0.59 to 0.08)	0.134	-0.16 (-0.65 to 0.32)	0.508		-0.21 (-0.44 to 0.03)	0.083
**Log sTNF-RII**	**0.31 (0.11 to 0.52)**	**0.002**	0.09 (-0.10 to 0.28)	0.337	0.25 (0.05 to 0.45)	0.014		-0.01 (-0.14 to 0.12)	0.866
**Log VCAM**	**0.42 (0.08 to 0.76)**	**0.014**	0.11 (-0.23 to 0.45)	0.530	0.16 (-0.19 to 0.51)	0.367		0.10 (-0.13 to 0.33)	0.392
**Log sCD163**	0.18 (-0.19 to 0.55)	0.333	-0.15 (-0.48 to 0.17)	0.348	-0.30 (-0.66 to 0.05)	0.095		0.07 (-0.13 to 0.28)	0.489
**Log d-dimer**	0.13 (-0.22 to 0.48)	0.460	0.11 (-0.22 to 0.43)	0.524	0.07 (-0.28 to 0.41)	0.707		**0.23 (0.07 to 0.39)**	**0.005**
**Log sCD14**	0.07 (-0.08 to 0.22)	0.331	**0.15 (0.01 to 0.29)**	**0.040**	0.07 (-0.10 to 0.25)	0.411		0.00 (-0.08 to 0.08)	0.996
**Log LBP**	**0.19 (0.06 to 0.33)**	**0.005**	**0.20 (0.06 to 0.34)**	**0.006**	**0.18 (0.02 to 0.35)**	**0.030**		-0.02 (-0.12 to 0.09)	0.759
**Log IL-6**	-0.23 (-0.85 to 0.39)	0.472	0.00 (-0.54 to 0.53)	0.987	0.03 (-0.55 to 0.61)	0.922		-0.17 (-0.50 to 0.16)	0.314
**Log hsCRP**	0.41 (-0.46 to 1.3)	0.355	NT		0.47 (-0.43 to 1.4)	0.305		-0.18 (-0.64 to 0.27)	0.430
**Log HbA1c**	NT		NT		-0.04 (-0.30 to 0.22)	0.759		**-0.17 (-0.30 to -0.04)**	**0.010**

^1^CI, Confidence Interval; NT, Not tested.

NB Sex and daily injecting drug use were included as covariates in the analysis but did not significantly affect the parameters shown (not shown).

Bold values indicate those which are statistically significant (p<0.05).

### Impact of DAA therapy on immune biomarkers in PWID with HCV

All participants in the PWID/HCV+ cohort initiated DAA therapy with sofosbuvir/velpatasvir and achieved sustained virol response at the end of treatment (EOT). HCV RNA was not detected in any participant between the EOT and 48wk timepoints (data not shown). Analysis of changes in immune biomarkers over time after DAA therapy indicated that levels of CXCL10 and VCAM, which were both elevated in PWID/HCV+ prior to treatment, were significantly reduced by DAA therapy (p<0.05 for all timepoints as compared to pre-treatment values, **Supplementary Table 2** and **Figure 3**). Comparison of post-DAA biomarker levels in PWID/HCV+ with PWID/HCV- participants revealed similar levels of CXCL10 and VCAM at EOT and 48 wk timepoints, implying a complete reversal of HCV-associated effects by DAA therapy (**Table 4**, **Figure 2**). sTNF-RII levels were reduced in PWID/HCV+ participants at the EOT but not 48wk post-DAA timepoints (p=0.002 and 0.453, respectively, compared with pre-treatment; **Supplementary Table 2**), and 48wk timepoint levels remained significantly elevated as compared to the PWID/HCV- participants (p=0.014, **Table 4**). In contrast, elevated levels of the endotoxin-binding factor LBP in PWID/HCV+ participants were not significantly altered by DAA therapy (p≥0.85, **Supplementary Table 2**) and remained higher than PWID/HCV- individuals at both post-treatment timepoints (p=0.006 and 0.030 at EOT and 48 weeks respectively, **Table 4**).

Regarding myeloid cell activation markers, DAA therapy was associated with a decrease in sCD163 (p<0.001 for EOT and 48wks, **Supplementary Table 2**) despite levels not being significantly different to PWID/HCV- at baseline, whilst sCD14 was significantly elevated in PWID/HCV+ at EOT (p=0.039 compared to pre-treatment, **Supplementary Table 2**) and was significantly higher than PWID/HCV- (p=0.040, **Table 4**). Other immune biomarkers measured including hsCRP, IL-6, HbA1c and d-dimer which were not different between PWID/HCV- and PWID/HCV+ prior to treatment were also not altered following DAA therapy. These analyses were adjusted for daily injecting drug use, which was a missing parameter in some PWID participants (**Table 2**). Thus modelling analyses were repeated without adjustment for this parameter to allow all participants to be included, which yielded very similar results (not shown). Taken together, these analyses suggest that DAA-mediated HCV cure reverses some elements of immune and endothelial activation induced by HCV viremia, but certain biomarkers of inflammation (i.e. sTNF-RII) and LPS signalling/regulation (i.e. LBP) are not fully reversed whilst others (i.e. sCD14) are actually increased by DAA therapy and remain elevated in PWID/HCV+ despite DAA-mediated HCV elimination.

## Discussion

Curative DAA therapy for HCV prevents substantial morbidity and mortality, but it is unclear whether DAA therapy leads to a complete reversal of HCV-related immune alterations and thus related pathologies. We identified elevated levels of numerous soluble biomarkers associated with chronic disease risk in PWID, compared to those with no history of injecting drug use, including inflammatory and monocyte activation biomarkers. PWID with chronic HCV exhibited elevated levels of additional parameters including biomarkers of endothelial activation and only some of these parameters were normalised by curative DAA therapy. These data suggest PWID may be at increased risk and thus should be closely monitored for the development of inflammatory morbidities. Furthermore, PWID with a history of HCV infection may possess additional and unique immunological alterations which persist after HCV cure and may potentiate the development of chronic diseases.

The specific biomarkers identified to be elevated in PWID with no history of HCV included the inflammatory biomarkers sTNF-RII and IL-6 and markers of myeloid cell activation (sCD14 and sCD163). These findings are consistent with previous observations in people who use drugs (37–40) and suggest a persistent state of inflammation and myeloid cell activation. Elevated levels of these biomarkers are associated with increased risk and severity of cardiovascular disease and mortality in both the general population and in individuals with underlying chronic infections such as HIV (21–23, 41, 42), however their relationship with chronic disease risk and mortality in PWID with and without HCV is not known and warrants further investigation.

Elevated levels of these inflammatory markers in PWID may be driven by a number of behavioural and sociodemographic factors including, but not limited to, diet, tobacco and alcohol use (32% of PWID in the present study reported using alcohol ≥4 times/week). Tobacco use and moderate to heavy alcohol consumption are associated with heightened inflammation (28) and these risks are prevalent in PWID populations (43). Similarly, there is a well-established link between diet and systemic inflammation (29, 44) and substance use is known to affect diet and compromise nutrition, which is potentiated by factors including unstable housing (45). There are also mechanisms directly related to injecting drug use itself such as inflammatory and immunomodulatory effects of the drugs injected including methamphetamine, opioids and accompanying filler agents (46–50). Injury and infection related to injection site and injection practices can result from contaminated injecting equipment and/or the introduction of pathogens, foreign bodies and skin contaminants during the process of injecting (25, 27, 51). Over 60% of PWID report a history of skin and soft tissue infections with subcutaneous/intradermal injecting practices and vascular damage associated with injecting contributing to this risk (26). Injection frequency and duration correlate positively with markers of immune activation including d-dimer and sCD14 (39), whilst methamphetamine users with HIV who injected the drug exhibited elevated levels of numerous inflammatory biomarkers (including IL-6, sTNF-RI and sCD163) compared to those who did not inject (52). Together, these findings support a direct contribution of drug injection to systemic inflammation in PWID irrespective of HCV status, and more research is needed to characterise these risks and determine their long-term health implications.

PWID in the present study also exhibited substantially elevated levels of HbA1c, which is a well validated indicator of type 2 diabetes risk, diabetic complications and cardiovascular disease (53). These metabolic alterations are likely associated with heightened inflammation but may also be influenced by lifestyle-related differences such as diet which is often altered in PWID (45). These data are consistent with the well documented association of injecting drug use with poor health outcomes and suggest inflammatory and cardiometabolic alterations may predispose them to increased risk of chronic inflammatory diseases such as cardiovascular disease and diabetes. Future studies are warranted to better characterise diabetes prevalence in PWID and identify contributing risk factors.

Analysis of the impact of active HCV infection in PWID revealed a further elevation of sTNF-RII levels as compared to never-infected PWID, as well as significant increases in plasma levels of CXCL10, VCAM and LBP in PWID/HCV+, consistent with previous findings (8, 9, 12, 17, 54). We did not detect an increase in inflammatory factors previously associated with chronic HCV such as sCD163, sCD14 or IL-6 in our cohort of PWID with HCV, potentially due to the pre-existing elevation of these parameters observed in all PWID. However, we did observe a significant reduction in sCD163 levels following DAA initiation, implying viremic HCV was contributing to heightened sCD163 levels and by association heightened macrophage activation prior to DAA initiation.

DAA therapy was associated with normalisation of certain activation markers (e.g., CXCL10, VCAM) to levels comparable to uninfected PWID, suggesting these parameters are closely linked with HCV viremia and are thus largely reversed following HCV cure. Although DAA therapy initially reduced sTNF-RII, levels at 48 weeks post treatment were higher in PWID treated for HCV as compared to never-infected PWID. In contrast, LBP levels were not significantly reduced by DAA therapy and remained elevated as compared to HCV-uninfected PWID up to 48 weeks post HCV cure. LBP is primarily produced by hepatocytes and is involved in the inflammatory response to microbial products such as bacterial LPS. Elevated LBP observed here in PWID with current or past HCV infection may indicate impaired hepatocyte/liver function due to persistent tissue damage or may be due to enduring damage to the gut or other mucosal barriers, resulting in heightened translocation of bacterial products. The latter is consistent with elevated plasma LPS levels in untreated HCV (55) and is analogous to the compromised gut barrier integrity and heighten microbial translocation observed in chronic treated HIV infection, which is known to contribute to persistent immune activation and inflammatory comorbidities (56). Elevated LBP and LPS levels are associated with dietary glucose and diabetes (57), so the observed elevation in HbA1c and LBP in the present cohort may be indicative of metabolic alterations. Little is known regarding the clinical implications of elevated LBP levels in people with HCV, however in HIV/HCV co-infection it is associated with more advanced liver fibrosis (58) and in HIV, elevated LPS and LBP are linked with increased risk of cardiovascular disease and dementia (59, 60), supporting the clinical relevance of these findings. Follow-up studies are required to identify the relationship between persistently elevated levels of inflammatory factors identified here and clinical outcomes in individuals after HCV cure, especially in PWID.

Interestingly, we also observed a significant increase in sCD14 levels in PWID with HCV after 12 weeks of DAA therapy consistent with previous finding (13), although this was normalised after 48 weeks. This may be related to transient immune reconstitution induced by viral suppression and resultant increase in myeloid cell activity, however this remains to be explored. Consistent with the well-established relationship between inflammation and impaired liver function, all biomarker analysed here (except HbA1c) were significantly associated by multivariable regression analysis with indices of liver function including AST, ALT and the AST to platelet ratio index (APRI) score (data not shown), the latter being a surrogate marker of liver disease in HCV (61). People treated for HCV with DAA therapy are at heightened risk of mortality related not only to liver disease but also extrahepatic cancers and circulatory diseases, and the presence of comorbidities further increases mortality risk (7). The findings presented here of a persistent inflammatory state in individuals post HCV cure may therefore be relevant to these long term health outcomes and it would be of interest to determine whether these biomarkers were associated with morbidity and mortality post HCV cure.

The limitations of this study include the modest sample size due to the selection criteria and sample availability at the required timepoints and a lack of data pertaining to lifestyle factors such as body mass index, level of physical activity, as well as medical history and current medications. A longer duration of HCV viremia may heighten the immune changes measured here due to more extensive immune activation/exhaustion and a greater accumulation of cellular and tissue damage; this parameter could not be controlled for since time of infection is difficult to determine. Injection practices were not assessed in this analysis but would be useful to include in future studies. Finally, we had limited demographic data available in our never infected community control participants which precluded formal investigation of behavioural factors which may contribute to the observed biomarker differences. Given their influence on the parameters measured here, future studies are warranted to specifically assess the contribution of behavioural factors to chronic inflammatory disease risk.

## Conclusions

These data add to a growing body of evidence that curative DAA therapy may not completely reverse immunological changes included by HCV infection, and biomarkers relevant to the development of chronic morbidities may remain elevated in some individuals. This study also highlights the particular vulnerability of PWID with HCV to these effects given the overlapping impact of injecting drug use and related lifestyle factors on immunological changes relevant to the development of chronic inflammatory diseases. These findings suggest ongoing clinical management of PWID with a history of HCV infection should include heightened vigilance for chronic inflammatory diseases even after curative HCV treatment.

## Data availability statement

The datasets used and/or analysed during the current study are available from the corresponding author on reasonable request.

## Ethics statement

Written informed consent to participate in this study was obtained from all participants. This work was approved by the Alfred Hospital Research and Ethics Committee (Melbourne, Australia).

## Author contributions

AH: Conceptualization, Formal analysis, Funding acquisition, Investigation, Resources, Writing – original draft. NV: Formal analysis, Writing – original draft. SE: Investigation, Writing – review & editing. BH: Data curation, Project administration, Validation, Writing – review & editing. IB: Resources, Writing – review & editing. LN: Data curation, Project administration, Writing – review & editing. DP: Formal analysis, Writing – review & editing. PD: Resources, Writing – review & editing. AD: Resources, Writing – review & editing. AT: Resources, Writing – review & editing. AJ: Conceptualization, Supervision, Writing – review & editing. MH: Resources, Writing – review & editing. RS-D: Data curation, Formal analysis, Supervision, Writing – original draft. JD: Conceptualization, Funding acquisition, Resources, Supervision, Writing – original draft.
