# Supplementary material for: Injecting drug use and hepatitis C virus infection independently increase biomarkers of inflammatory disease risk which are incompletely restored by curative direct-acting antiviral therapy
Source: Front Immunol. 2024 Feb 14;15:1352440. doi: 10.3389/fimmu.2024.1352440 (PMC10899672; doi:10.3389/fimmu.2024.1352440)
Supplement: Supplementary file 1 [file DataSheet_1.docx]

|  | **Sex: Male** | | **Age (10/years)** | | **Injecting Drugs past month** | |
| --- | --- | --- | --- | --- | --- | --- |
|  | *Beta (95% CI)^1^* | *p-value* | *Beta (95% CI)^1^* | *p-value* | *Beta (95% CI)^1^* | *p-value* |
| **Log CXCL10** | 0.14 (-0.23 to 0.51) | 0.447 | -0.10 (-0.32 to 0.11) | 0.347 | 0.09 (-0.27 to 0.45) | 0.612 |
| **Log sTNF-RII** | 0.01 (-0.17 to 0.19) | 0.898 | 0.03 (-0.07 to 0.14) | 0.540 | **0.26 (0.09 to 0.44)** | **0.004** |
| **Log VCAM** | 0.10 (-0.17 to 0.36) | 0.467 | 0.01 (-0.15 to 0.16) | 0.915 | -0.01 (-0.27 to 0.25) | 0.919 |
| **Log sCD163** | 0.02 (-0.36 to 0.41) | 0.911 | -0.05 (-0.27 to 0.18) | 0.677 | 0.15 (-0.22 to 0.53) | 0.415 |
| **Log D-Dimer** | -0.36 (-0.74 to 0.02) | 0.062 | **0.23 (0.01 to 0.45)** | **0.043** | **0.51 (0.14 to 0.88)** | **0.008** |
| **Log sCD14** | -0.10 (-0.26 to 0.07) | 0.243 | 0.02 (-0.08 to 0.12) | 0.661 | **0.18 (0.02 to 0.34)** | **0.031** |
| **Log LBP** | -0.16 (-0.35 to 0.04) | 0.121 | 0.01 (-0.10 to 0.13) | 0.810 | 0.02 (-0.17 to 0.21) | 0.826 |
| **Log IL-6** | -0.06 (-0.70 to 0.59) | 0.863 | -0.02 (-0.40 to 0.35) | 0.904 | 0.59 (-0.04 to 1.2) | 0.065 |
| **Log hsCRP** | -0.30 (-1.1 to 0.48) | 0.447 | 0.18 (-0.28 to 0.63) | 0.440 | 0.55 (-0.20 to 1.3) | 0.148 |
| **Log HbA1c** | 0.03 (-0.31 to 0.37) | 0.874 | -0.03 (-0.22 to 0.15) | 0.716 | **0.65 (0.35 to 0.96)** | **<0.001** |
| ^1^CI = Confidence Interval  Analyses were adjusted for sex, age and injecting drug use in the past month. | | | | | | |

**Supplementary Table 1**: Multivariable Liner Regression analysis of inflammatory biomarkers in PWID in reference to community controls adjusting for recent injecting drug use.

**Supplementary Table 2:** Generalised Estimating Equation analysis of inflammatory biomarkers in HCV+ PWID post- compared to pre-DAA therapy.

|  | **Timepoint (ref: pre-treatment)** | | |  |  | **Age (10/years)** | |
| --- | --- | --- | --- | --- | --- | --- | --- |
|  | EOT | | 48wk post-DAA | |  |  |  |
|  | *Beta (95% CI)^1^* | *p-value* | *Beta (95% CI)^1^* | *p-value* |  | *Beta (95% CI)^1^* | *p-value* |
| **Log CXCL10** | **-0.76 (-0.98 to -0.54)** | **<0.001** | **-0.67 (-1.1 to -0.25)** | **0.002** |  | -0.21 (-0.44 to 0.03) | 0.083 |
| **Log sTNF-RII** | **-0.22 (-0.36 to -0.08)** | **0.002** | -0.06 (-0.23 to 0.10) | 0.453 |  | -0.01 (-0.14 to 0.12) | 0.866 |
| **Log VCAM** | **-0.31 (-0.43 to -0.20)** | **<0.001** | **-0.26 (-0.49 to -0.03)** | **0.024** |  | 0.10 (-0.13 to 0.33) | 0.392 |
| **Log sCD163** | **-0.34 (-0.49 to -0.18)** | **<0.001** | **-0.49 (-0.70 to -0.27)** | **<0.001** |  | 0.07 (-0.13 to 0.28) | 0.489 |
| **Log D-Dimer** | -0.02 (-0.24 to 0.20) | 0.828 | -0.06 (-0.36 to 0.23) | 0.671 |  | **0.23 (0.07 to 0.39)** | **0.005** |
| **Log sCD14** | **0.07 (0.00 to 0.14)** | **0.039** | 0.00 (-0.11 to 0.11) | 0.973 |  | 0.00 (-0.08 to 0.08) | 0.986 |
| **Log LBP** | 0.00 (-0.06 to 0.07) | 0.906 | -0.01 (-0.11 to 0.09) | 0.849 |  | -0.02 (-0.12 to 0.09) | 0.759 |
| **Log IL-6** | 0.22 (-0.13 to 0.58) | 0.213 | 0.26 (-0.15 to 0.66) | 0.215 |  | -0.17 (-0.50 to 0.16) | 0.314 |
| **Log hsCRP** | NT |  | 0.06 (-0.63 to 0.75) | 0.861 |  | -0.18 (-0.64 to 0.27) | 0.430 |

^1^CI = Confidence Interval

NT: Not tested, EOT: End of treatment, DAA: Direct acting antivirals.

NB Sex and daily drug injection were included as covariables in the analysis but were not significantly associated with the above parameters.
